# Acute Schizophrenia-like Psychotic Disorder Against the Background of COVID-19

**DOI:** 10.3390/medicina61020298

**Published:** 2025-02-08

**Authors:** Lidia Bravve, Maria Kaydan, Georgy Kostyuk

**Affiliations:** 1 Psychiatric Hospital No. 1 Named After N.A. Alexeev of the Department of Health of Moscow, 115191 Moscow, Russia; kaydan.maria@yandex.ru (M.K.);; 2 Department of Mental Health, Faculty of Psychology, M. V. Lomonosov Moscow State University, 119991 Moscow, Russia; 3 Department of Psychiatry, Federal State Budgetary Educational Institution of Higher Education Russian Biotechnological University, 125080 Moscow, Russia; 4 Department of Psychiatry and Psychosomatics, I. M. Sechenov First Moscow State Medical University (Sechenov University), 119435 Moscow, Russia

**Keywords:** acute schizophrenia-like psychotic disorder, COVID-19, schizophrenia, psychosis treatment

## Abstract

*Background and Objectives*: Research in this area focuses on acute schizophrenia-like psychotic disorder, as more than half of cases progress to a chronic course, manifesting as schizophrenia or schizoaffective disorder. Research has shown a link between viral infection and the onset of psychosis, and the influence of viruses on the clinical course of the disease is also being studied. Consequently, in cases where this type of psychosis co-occurs with a viral illness, there is a compelling rationale for identifying commonalities in both treatment and outcome. The ongoing global pandemic of COVID-19 provides a unique opportunity to assess these changes. The aim of this study is twofold: first, to examine the clinical characteristics of acute schizophrenia-like psychotic disorder in the context of the pandemic, and second, to analyze therapeutic interventions and outcomes. *Materials and Methods*: A non-invasive observational study was conducted in which 310 patients with acute schizophrenia-like psychotic disorder admitted as inpatients to a psychiatric hospital were divided into two groups according to the presence of COVID-19 (group I—F23.2 with COVID-19): 222 patients; Group II—F23.2 without COVID-19: 88 patients). After discharge, the patients in both groups were clinically followed in outpatient treatment for 36 months. *Results*: The results showed that acute schizophrenia-like psychotic disorder associated with COVID-19 was characterized by a greater severity of productive symptoms and the inclusion of the pandemic theme in psychotic symptoms. These patients were given higher doses of antipsychotic medication. *Conclusions*: The outcome of this type of psychosis is consistent, irrespective of the infection suffered at the onset of the disease, and is characterized by a chronic course with a predominant transition to the schizophrenic process.

## 1. Introduction

Acute schizophrenia-like psychotic disorder is an acute mental disorder in which the psychotic symptoms are comparatively stable and justify a diagnosis of schizophrenia, but have lasted for less than about one month [1]. Such psychoses can occur against the background of various exogenous stressors, such as viral diseases, alcohol abuse, or traumatic brain injury. The likelihood of people contacting psychiatrists in this condition has increased, both due to the increased frequency of acute transient psychotic disorder (ICD-10 F23) in the population, ranging from 3.9 to 9.6 cases per 100,000 people per year [2,3,4,5], and due to the duration of the pandemic, which has lasted more than three years. In total, more than 700 million people have been infected with some form of coronavirus infection [6]. Acute psychotic conditions, which are considered in the 10th revision of the International Classification of Diseases (ICD-10) under the title “Schizophrenia, schizotypal and delusional disorders”, are characterized by a more favorable prognosis when compared with schizophrenia [3,7]. On the contrary, in the current version of the recommendations at the time of writing (ICD-11), the subtitle of acute psychotic disorders has been subject to revision. An “acute schizophrenia-like psychotic disorder” is no longer classified as an acute schizophrenia-like psychotic disorder, but rather is encoded as an “unspecified primary psychotic disorder” [8].

The reclassification of acute schizophrenia-like psychoses within the framework of the International Statistical Classification of Diseases and Related Health Problems is likely to be based on the findings of long-term case studies, where the initial diagnosis of acute schizophrenia was replaced by schizophrenia for an extended period [9,10,11], in comparison with the patients diagnosed with acute polymorphic psychotic disorder without symptoms of schizophrenia who had only a 34% chance of a change in diagnosis [12], and the probability of a change in diagnosis was significantly higher. Consequently, an acute schizophrenia-like psychotic disorder is characterized as a condition that creates a significant risk of developing schizophrenia, though this transition is not inevitable. This emphasizes the need for intensive monitoring for this particular psychosis, since it represents a high-risk category for the transition to a chronic course of the schizophrenic process.

At the same time, it is appropriate to consider the topic of acute schizophrenia-like psychotic disorders from the perspective of the impact of acute respiratory viral infections, particularly COVID-19, on the progression of psychosis itself and its consequences. Numerous studies have been conducted on the clinical and psychopathological features of such psychoses. However, the existing literature is mainly limited to descriptions of the prevailing symptoms and a relatively short follow-up period (typically ranging from 2 to 8 weeks) [13,14,15,16,17,18,19,20,21,22]. To date, no studies have been conducted using a complex analysis with a long-term follow-up. Concurrently, numerous authors present data on the pathoplastic effect of infection on the course and outcome of psychoses [23,24,25,26,27]; it is assumed that this may be relevant to the selection of treatment strategies, as well as the general prognosis of the disease.

Nevertheless, the study of the features of psychopharmacotherapy in the simultaneous treatment of acute schizophrenia-like psychotic disorders and COVID-19 is limited to the assessment of the interaction between antipsychotic and antiviral medications, as well as their respective general properties [28,29,30]. According to the data of individual case histories, the application of atypical antipsychotics as psychopharmacotherapy was recorded [31,32,33,34,35] in minimal or average therapeutic doses [36,37,38]. Additionally, the prescription of benzodiazepines [20,39] and mood-stabilizing agents [40,41] was recorded. In some cases, the administration of drug therapy was supplemented by electroconvulsive therapy [42,43]. Thus, some features of the administered therapy of acute schizophrenia-like psychoses against the background of COVID-19 remain unstudied, and studies in this area will significantly complement and expand the previously obtained data on the treatment of schizophrenic spectrum disorders. Meanwhile, COVID-19 is a universal model for studying the effect of infection on the course of psychosis.

Schizophrenia-like psychoses with a COVID-19 course have been considered in a number of review articles, where the peculiarities of the sociodemographic characteristics of these patients and the clinical and dynamic indicators of the course of this disorder have been studied [15,44,45,46]. Therefore, the pandemic has provided an optimal opportunity to conduct a comprehensive study of how schizophrenia-like psychoses have been modified by exogenous damage, such as an acute respiratory infection. In addition to the ‘direct’ acting of coronaviruses, in the form of dysfunction of the blood–brain barrier, it should be remembered that the following case is also indicative [47,48]. The pandemic has become a significant source of stress for many people, primarily due to the stringent lockdown measures taken and the fear of immediate infection that these measures have created [49,50,51]. The existing literature suggests that stress plays a significant role in the development of psychosis. This hypothesis is supported by the occurrence of reactive psychosis in the absence of a prior infection with COVID-19 during the pandemic [52,53]. The objective of this study was to investigate the clinical manifestations of acute schizophrenia-like psychotic disorder in the ongoing pandemic of COVID-19, analyze therapeutic interventions, and monitor possible outcomes. However, the study did not make it a point to differentiate between the effects of infection and stressors, nor did it examine their influence on the configuration of acute schizophrenia-like psychotic disorder. It is assumed that symptoms of psychosis are subject to change when co-occurring with viral infection, since endogenous psychosis occurs against a background of exogenous damage. And it is likely that the substrate of psychotic experiences is also subject to change. This study was conducted to examine the clinical features of acute schizophrenia-like psychotic disorder in the context of the ongoing pandemic of COVID-19, analyze therapeutic interventions, and monitor possible outcomes.

## 2. Materials and Methods

### 2.1. Materials

An observational, prospective, and non-interventional study was conducted involving 310 patients (135 men and 175 women, with an average age of 28.8 ± 6.6) who were admitted to a psychiatric clinic in the period from 2020 to 2022, with follow-up care for 36 months.

The study’s objective was to address specific problems, and patients with acute schizophrenia-like psychotic disorder against the background of COVID-19 and acute schizophrenia-like psychotic disorder without the aforementioned background were selected for the study. This study was conducted on a cohort of patients admitted to a psychiatric hospital. The admission was urgently indicated due to the patients’ psychiatric statuses. The possibility of monitoring patients with acute schizophrenia-like psychotic disorder against the background of the virus was associated with the organization of special care units for patients with mental disorders on the basis of a psychiatric hospital and simultaneous course of the virus.

The study was conducted in accordance with the ethical principles of the World Medical Association’s Declaration of Helsinki of 1964 (last revision in 2013) and approved by the Local Ethics Committee (28 October 2020, Protocol No. 2).

The inclusion criteria in the study were as follows:

- no previous visits to a psychiatrist;
- the clinical picture of acute psychotic disorder meeting the criteria of “acute schizophrenia-like psychotic disorder” (F23.2) according to the International Classification of Diseases, Tenth Revision (ICD-10);
- age between 18 and 60 years;
- voluntary informed written consent to participate in the study.

The following criteria were used to determine non-inclusion:

- no systematic use of psychoactive substances and alcohol in the past medical histories (it means substance use disorder (mild)), no examination by a narcologist, and no use of psychoactive substances during the 6 months prior to admission;
- a somatic disease in the decompensation stage (in accordance with the established medical protocols of the hospital, all patients were subjected to consultation by a general practitioner, a neurologist, and an infectious disease specialist);
- the severe course of the novel coronavirus infection (dyspnoea—a feeling of air shortage, tightness in the chest area, shortness of breath or tachypnoea, cyanosis/acrocyanosis, SpO_2_ ≤ 93%; the treatment of severe cases of the COVID-19 was carried out in accordance with the clinical recommendations that were adopted in our country by means of admission to an intensive care unit. The severe course of the disease is frequently associated with the decompensation of concomitant somatic pathologies and a decrease in the activity of liver isoenzymes that are involved in the metabolism of antipsychotic agents).
- pregnancy and lactation.

**Figure 1:**
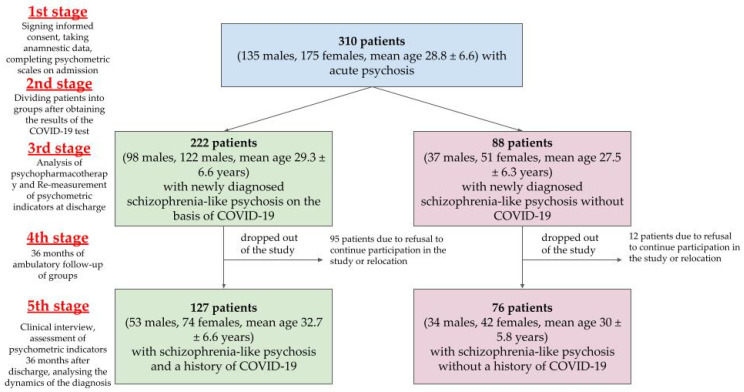
Study diagram.

The study diagram is shown in Figure 1.

In the first stage of the study, 310 patients (135 men and 175 women, with an average age of 28.8 ± 6.6 years) with acute schizophrenia-like psychosis were selected. Upon admission to the emergency department, all patients underwent a laboratory test for coronavirus infection, which was prepared within 1 to 3 days. During the first week of admission, the patients were invited to participate in the study by giving voluntary informed written consent. Thereafter, psychiatrists conducted clinical interviews, collected objective medical history data, psychometrically assessed the conditions, and analyzed the patients’ medical records.

In the second stage, the patients were divided into two groups depending on the results of laboratory testing for the virus that causes pneumonia, known as “coronavirus disease 2019” (hereinafter referred to as “COVID-19”).

Group I—F23.2 with confirmed cases of the novel coronavirus: 222 patients (98 men, 122 women, with an average age of 29.3 ± 6.6 years) with a recent onset of acute schizophrenia-like psychotic disorder coinciding with the presence of the novel coronavirus.

Group II—F23.2 without confirmed cases of the novel coronavirus: A total of 88 patients (37 men, 51 women, with an average age of 27.5 ± 6.3 years) with recently diagnosed acute schizophrenia-like psychotic disorder and with no past medical history of COVID-19 were included in the study.

The third stage of the study was conducted at the time of hospital discharge. At this stage, the analysis of psychopharmacotherapy and the re-measurement of psychometric indicators was conducted.

The fourth stage of the study consisted of outpatient observation of patients for a period of 36 months. All patients were discharged to be followed up with by a psychiatrist in a neuropsychiatric dispensary. However, 95 patients from the group with confirmed cases and 12 patients from the group without confirmed cases dropped out of the study due to the refusal to continue participating or relocation. After excluding individual patients, the groups were maintained in the following composition:

Group I—F23.2 with a past medical history of COVID-19 (127 observations, 53 men, 74 women, 32.7 ± 6.6 years old).

Group II—F23.2 without a past medical history of COVID-19 (76 patients, 34 men, 42 women, 30 ± 5.8 years old).

In the fifth stage, after a 36-month period of outpatient care, an additional meeting with a research physician was conducted. This meeting included a clinical interview, the measurement of psychometric indicators, and an analysis of the diagnosis dynamics.

The attending physician of the department prescribed psychopharmacotherapy, while an infectious disease specialist prescribed antiviral therapy. The authors of the study did not interfere with the treatment regimen but conducted an analytical study of the treatment.

### 2.2. Methods

This study used a wide range of methodologies, encompassing clinical (data obtained from clinical interviews and objective medical records), clinical follow-up, psychometric (using the Positive and Negative Syndrome Scale [54] and the National Early Warning Score-2 [55]), paraclinical (including laboratory and instrumental studies), and statistical methods. NEWS-2 included six simple physiological parameters that formed the basis of the scoring system: respiration rate, oxygen saturation, systolic blood pressure, pulse rate, a level of consciousness or new mental confusion, and temperature.

The psychiatric diagnosis was confirmed according to the International Classification of Diseases, Tenth Revision (ICD-10) criteria. For the final expert assessment, each patient was examined by a group of psychiatrists, including two authors of this study and one head of the department.

On the first day of admission to the hospital, a psychiatrist assessed a patient’s mental state, obtained medical history data from relatives, and reviewed all available medical records (including records of advice from an emergency department psychiatrist, an admission department psychiatrist, and previous hospital discharges, if applicable). Then, the same doctor completed the psychometric data for each individual patient. After that, the same psychiatrist conducted a longitudinal clinical follow-up.

The virus was identified by a positive result of laboratory testing for the presence of SARS-CoV-2 virus RNA using nucleic acid amplification technologies. It should be noted that all patients admitted to the hospital underwent laboratory testing for the new strain of the virus, whilst all study participants underwent computed tomography of the chest organs to assess the development of pneumonia as a complication of the infection. This assessment was conducted using the General Electric 1.5T CT scanner.

The statistical processing of the obtained data was carried out using the Jamovi-2.2.5 program. To analyze the relationships of the qualitative features, the chi-square test for conjugacy tables was calculated. The statistical significance of differences in groups of quantitative variables was assessed using the nonparametric Mann–Whitney U test for two independent samples and the H Kruskal–Wallis test for independent samples. Differences with a *p*-value less than 0.05 were considered statistically significant.

Despite the observational nature of this study, the required sample size was adequately calculated. One of the indicators of interest was the frequency of inclusion of the pandemic topic in the content of psychotic experiences. According to various scholars, this range varies considerably, from 0% to 70%, reflecting significant variability. The calculation for the qualitative endpoint was carried out using the formula, Sample size(*n*) = (z_1−a/2_)^2^*(p)(q)/(d)^2^, where *n* is the required sample size, z_1−a/2_ is the critical z value of the distribution for the corresponding level of an error of the first kind (1.96 for 0.05), p is the expected frequency of the event, and q = 1 − p, d is the adequate accuracy of the estimate [56]. Assuming a pandemic topic inclusion rate of 30% (0.3) and a confidence interval of a fraction of 10% (0.1), 81 patients were required, excluding dropouts (per group). The selection of this group size was made in light of the uncertainty associated with the true frequency of the indicator under study, combined with the need for the collection of additional indicators. Thus, this group size was designated as the minimum required, with efforts made to increase the number of study participants.

### 2.3. Characteristics of COVID-19

In this study, the diagnosis of 222 patients was confirmed as positive for the new strain of COVID-19, commonly called “coronavirus disease 2019” (henceforth referred to as “COVID-19”). The data set revealed that 57% (*n* = 126) of the patients exhibited symptoms consistent with an acute respiratory viral infection, 31% (*n* = 69) were diagnosed with pneumonia without respiratory failure, and 12% (*n* = 27) exhibited symptoms consistent with both an acute respiratory viral infection and pneumonia.

The infection caused by the novel virus was found to range from mild to moderate in severity, as per the clinical recommendations. Mild severity was characterized by an increase in body temperature not exceeding 38.5 °C and an absence of dyspnoea at rest, but it may occur during exertion, SpO_2_ > 95%. The moderate degree of severity was characterized by an increase in body temperature above 38.5 °C and absence of dyspnoea at rest, but it may occur during exertion (screaming/crying), SpO_2_ ≤ 95%.

According to the findings of computed tomography, in 57 cases of patients diagnosed with pneumonia, less than 25% of the lung volume was affected. In 12 cases, 25 to 50% of the lung parenchyma was affected. A mean score on the NEWS-2 scale for patients with COVID-19 upon admission was 0.5 ± 0.6, reflecting satisfactory organ functioning and a low risk of complications. The most common somatic symptoms were fever (75%) and cough (51%), while diarrhea (3%) and nausea or vomiting (1%) were among the least common. The average duration of treatment in the infectious diseases department was 12.2 ± 5 days, after which the patients were transferred to the general psychiatric department to stabilize their mental state. It is noteworthy that, in special care units, the patients received both psychopharmacotherapy and antiviral treatment concurrently.

The administration of etiotropic therapy was carried out in accordance with the methodological recommendations stipulated by the local Ministry of Health at the time of material procurement [57]. The most frequently prescribed drug was umifenovir (33% of cases), followed by hydroxychloroquine (28%), favipiravir (21%), and imidazolyl ethanamide pentandioic acid (18%). The dosages of etiotropic therapy drugs in all patients corresponded to these guidelines: Hydroxychloroquine was prescribed at a dosage of 400 mg, administered twice on the first day, followed by 200 mg, administered twice a day for six days. Favipiravir was prescribed at a dosage of 1800 mg, administered twice a day on the first day, followed by 800 mg, administered twice a day from day 2 to day 10. In cases of patients weighing more than 75 kg, umifenovir was prescribed at a dosage of 200 mg, administered four times a day for a period of five to seven days. Imidazolyl ethanamide pentanedioic acid was used at a dosage of 180 mg, administered once a day for the initial three days of the disease, followed by a dosage of 90 mg, administered once a day for the subsequent four days. Antibacterial therapy was prescribed in 42% (92 patients) of cases, with azithromycin being the most frequently prescribed drug (53%), followed by levofloxacin (37%). Ceftriaxone and cefepime were used in rare cases. Glucocorticoid therapy and oxygen therapy were not prescribed in the study group.

## 3. Results

### 3.1. Clinical Characteristics of Patients upon Admission and During Hospital Treatment

Patients with newly emerging schizophrenia-like psychoses against the background of COVID-19 are significantly older (cf. the age of 28.5 ± 6.3 years at the time of the study) than patients without the infection (27.5 ± 6.3 years old, respectively) (Table 1). Furthermore, the onset of acute schizophrenia-like psychotic disorder during the course of infection was observed in individuals who maintained a satisfactory level of functioning, in contrast to those not infected. This observation was reflected in the higher rate of employment among patients with a diagnosis of schizophrenia due to the infection, at 40% compared to 11% among those who were not infected. In addition, a higher proportion of the patients diagnosed with virus-related psychosis were engaged in higher education at the time of admission, in comparison to those who were not infected (15% versus 8%, respectively). The two groups turned out to be comparable with regard to education and marital status.

A comparative analysis of the clinical picture of psychoses revealed that in the presence of concomitant cases of COVID-19, the symptoms were more severe and marked, that is, the condition was more critical. This finding was confirmed by an analysis of the Positive and Negative Syndrome Scale (PANSS) subscale of positive symptoms, which revealed that the mean scores exhibited a substantial discrepancy between the infected and non-infected groups (a mean score was 32.7 ± 5.0 vs. 29.3 ± 3.9). Furthermore, a significant discrepancy was found when comparing specific items in the Positive and Negative Syndrome Scale (PANSS) subscale of the positive disorders, namely P1 delusions (a mean score was 6.2 ± 0.7 vs. 5.6 ± 0.8), P2 conceptual disorganization (a mean score was 5.9 ± 0.8 vs. 5.2 ± 0.5), P3 hallucinations (a mean score was 5.5 ± 1.5 vs. 5.1 ± 0.8), and P5 grandiosity (a mean score was 3.6 ± 1.9 vs. 2.4 ± 1.5).

The impact of the COVID-19 topic on the clinical picture and the content of psychotic disorder in Group I patients was obvious. In contrast, in a cohort of patients not affected by the virus, there was no mention of the pandemic in all cases. In order to demonstrate the involvement of the pandemic topic in the content of psychotic disorder, the principal syndrome was first identified. The distribution of patients is presented below; 51% of cases were classified as hallucinatory–paranoid, 23% as delusional disorder (persistent), 10% as paranoid, and 17% as hallucinatory. In the case of persecutory delusions with auditory hallucinations (*n* = 113, 58% of men, 55% of women; the average age was 27.8 ± 5.9 years), the pandemic topic was implicated in delusions of exposure in 21% of cases (*n* = 24). Patients reported the belief that they were being subjected to “irradiation” by the virus through devices, with the presence of sensory automatisms along with the classical ideational automatisms. These were interpreted as the result of viral poison. Patients reported experiencing “burning” sensations in various areas of the body, as well as “burning” of organs and “constriction of the lungs” due to “irradiation” with the SARS-CoV-2 virus. In some cases (17%), motoric automatisms were also developed, in which patients froze without moving as a result of the “paralyzing” effects of the poison. Furthermore, in several cases, patients reported a sensation of “airlessness” in their lungs, a phenomenon that was also associated with the consequences of the disease. For instance, patients described their experience as “an air shortage in their body”, and cited a consumptive cough as evidence of their symptoms. Consequently, the somatic symptoms underwent a delusional interpretation and were incorporated into the delusional narrative. In delusional disorder (persistent) (*n* = 50, 19 men, 31 women; cf. the age of 33.8 ± 8.2 years), the topic of the pandemic was included in the content of psychotic disorder most often—in 78% of observations (*n* = 39). Patients considered themselves as the primary agents in the fight against the pandemic, perceiving themselves as “saviours of humanity”. They asserted that they had unique knowledge and abilities, including the capacity of “making a vaccine against the virus” (despite the lack of the required specialized education and skills). At the peak of acute antagonistic delusions, patients developed a oneiroid state (dream-like dissociative consciousness), where the topic of COVID-19 occupied a central place in the psychotic experiences. In the leading hallucinatory syndrome (37 patients, 8 men, 29 women; an average age was 26.2 ± 4.9 years), the pandemic topic was observed in 14% (*n* = 5) of cases and manifested by auditory hallucinations of a threatening nature. The discourse pertaining to the pandemic manifested in the form of death threats directed towards a patient or assertions that a patient’s relatives had contracted the disease (if they did not actually have the condition). In cases of the leading paranoid syndrome (*n* = 22, 13 men, 9 women; cf. the age of 26.6 ± 4.7 years) in 55% (*n* = 12), patients involved the topic of the pandemic in the delusional ideas—they believed that the persecutors deliberately infected them with COVID-19 in order to “seize their organs” or “kill them”. A third of the patients considered the medical staff to be intruders and viewed them as a part of a “criminal gang” that was in cahoots with the persecutors. In hypochondriacal delusions, patients consistently denied infection with COVID-19. Instead, they attributed their symptoms to a surreptitious concealment of an alternative diagnosis by healthcare professionals. Some of the patients expanded the delusional narrative and, after being admitted to the hospital, alleged that they had been administered antiviral medications that supposedly triggered mechanisms capable of disrupting organ function. Then they began to report discomfort, referring to symptoms such as “the throat moves”, “fish like crystals move in the brain”, “layers of the abdomen are removed”, “organs are cramping on my back”, “bones are mutating”, and “sticky plaque on my teeth”. In these cases of paranoid syndrome, somatic delusions with abnormal tactile perceptions were observed and interpreted as the consequences of drug therapy against COVID-19.

In both groups, antipsychotic medications were used to treat acute schizophrenia-like psychotic disorder (see Table 2 for details). In cases of psychotic disorder arising against the background of the pandemic, psychopharmacotherapy was prescribed in combination with the prescribed treatment for the virus.

Group I patients received antipsychotic therapy only in 44% (*n* = 98) of cases, while in Group II, those who were not diagnosed with COVID-19, this figure was 56% (*n* = 49). In half of the cases in the study groups, the main antipsychotic prescribed was haloperidol (47% vs. 57%), followed by zuclopentixol (20% and 24%), risperidone (16% and 11%), chlorpromazine (7% and 3%), olanzapine (7% and 2%), and ziprasidone (3% and 2%). The mean chlorpromazine equivalent (CPZ eq.) of antipsychotic drugs used to relieve psychotic disorder was significantly higher among Group I patients with COVID-19, at 330 ± 139 mg/day, compared to patients without infection, at 233 ± 92.2 mg/day. In addition, the mean doses of the drugs used also differed and were higher in patients with the infection, including haloperidol (a mean of CPZ eq. was 305 ± 83 vs. 218 ± 72.0 mg/day), zuclopentixol (a mean of CPZ eq. was 273 ± 97.3 vs. 219 ± 60.2 mg/day), and risperidone (a mean of CPZ eq. was 475 ± 56.7 vs. 390 ± 77.5 mg/day). Furthermore, chlorpromazine (*n* = 61 and *n* = 30) and tiapride (*n* = 63 and *n* = 19), prescribed primarily at night for sedation, were used as the second antipsychotic agent in both cohorts with similar regularity. Diazepam was used significantly less frequently to relieve psychomotor agitation or reduce anxiety levels during the acute phase of psychotic experiences—in 19% (*n* = 42) of cases with infection and, correspondingly, in 64% (*n* = 56) with psychoses without COVID-19 (χ^2^ = 58.3; *p* < 0.001). Diazepam was used exclusively during the initial days of admission, and there were no significant differences in the duration of administration between the groups. Anticholinergic anti-Parkinson agents were prescribed to 59% (*n* = 130) of patients with acute schizophrenia-like psychotic disorder against the background of the confirmed or suspected cases of the novel coronavirus (*n* = 130), 60% (*n* = 53), and among them, there was no documented past medical history of the virus. Trihexyphenidyl was the most commonly prescribed medication in both groups (31% and 41% of cases, respectively). A therapy using mood-stabilizing agents was added in 27% (*n* = 60) of acute schizophrenia-like psychotic disorder cases with viral presence and in 31% (*n* = 27) of cases without viral presence. No significant differences were observed in the frequency of prescriptions or the dosage of a drug used, valproic acid, and it was combined on the 7th–10th day of admission. In both groups, diagnosed with acute schizophrenia-like psychotic disorder, the efficacy of relief therapy was confirmed by a decrease in the overall Positive and Negative Syndrome Scale (PANSS) score by more than 25% upon discharge [58], with the same duration of treatment in a 24 h hospital (for patients diagnosed with COVID-19, the average duration of treatment was 28.1 ± 4.7 days, while for patients not diagnosed with COVID-19, it was 28.7 ± 3.6 days; *p* = 0.464).

### 3.2. Clinical Characteristics of Patients Under a Longitudinal Clinical Follow-Up

The follow-up was monitored after 36 months in 203 patients, including among Group I patients (127 observations, 53 men, 74 women, aged 32.7 ± 6.6 years old) with a new onset of acute schizophrenia-like psychotic disorder against the background of COVID-19, and in 64%, the disorder became chronic. In 49 cases (39%), a diagnosis of schizophrenia was established. In 25 cases (20%) was schizoaffective disorder, and in 6 (5%) was bipolar disorder (Table 3).

In the course of monitoring a cohort of 76 patients diagnosed with schizophrenia (34 men, 42 women, with an average age of 30 ± 5.8 years) with newly diagnosed acute schizophrenia-like psychotic disorder and no prior history of severe acute respiratory syndrome (SARS) CoV-2 infection, it was found that, in 74% of cases, the disorder became chronic. In 35 cases (46%), the diagnosis was changed to schizophrenia, and in 21 cases (28%), the disorder was diagnosed as schizoaffective.

As shown in Table 4, the average number of hospital admissions in 36 months in a 24 h hospital and the average number of admissions for 36 months in a day hospital are comparable for both groups after 36 months of longitudinal clinical follow-up.

Patients in both groups were equally likely to be referred to outpatient daycare centers. The same number of patients were referred to a day hospital for treatment with exacerbations of positive symptoms (29% vs. 28%) and joining depressive and anxiety symptoms (9% vs. 7%). In the early stages of the disease, patients suffering from COVID-19 were slightly less inclined to seek medical attention for symptoms of a more negative nature than those who did not contract COVID-19 (18% vs. 26%). This difference is probably explained by the following factor, namely that patients with acute schizophrenia-like psychotic disorder at the onset of the disease against the background of COVID-19 showed a less-pronounced shift in the emotional and volitional spheres compared to patients without infection. This finding is supported by the lower Negative Syndrome ScalePANSS (a mean score of 29.0 ± 7.1 vs. a mean score of 31.5 ± 7.2, respectively). The study revealed no statistically significant differences in total Positive and Negative Syndrome Scale (PANSS) (a mean score of 58.6 ± 21.9 vs. a mean score of 60.5 ± 16.1) and Positive syndrome scale PANSS (a mean score of 10.2 ± 7.3 vs. a mean score of 11.3 ± 9.2, respectively). However, the mean of the scores on the General Psychopathology Scale PANSS were found to be higher among patients diagnosed with COVID-19 compared to those without COVID-19 (a mean score of 19.6 ± 9.5 vs. a mean score of 17.8 ± 7).

In the 36-month period under consideration, approximately one-third of patients in both groups have developed occupational disability (27% vs. 29%). A comparison of the labor employment of the two groups of patients revealed no significant differences. However, the values indicated that the group of patients who contracted COVID-19 at the onset of the pandemic more frequently maintained employment without role demotion than patients without COVID-19 (17% vs. 1%). Concurrently with this indicator, a greater number of patients with COVID-19 were observed to have reduced qualifications and to have stopped working than patients without COVID-19 (19% vs. 1% and 24% vs. 9%). A comparable trend was observed in the context of university enrolment, with 9% of patients who had contracted COVID-19 continuing their study, while only 5% of patients without COVID-19 continued their study.

In summary, the clinical and dynamic indicators of patients who suffered from schizophreniform psychosis during the manifestation of the disease are consistent, irrespective of the presence of a concurrent respiratory infection. This is confirmed by the uniform distribution of diagnoses after 36 months of clinical observation and follow-up of patients, and the absence of differences in other indicators.

## 4. Discussion

The diagnosis of acute schizophrenia-like psychotic disorder is manifested by symptoms of schizophrenia that last less than one month. However, this acute schizophrenia-like psychotic disorder is often an “intermediate” state, as it is a manifestation of schizophrenia spectrum or affective disorders [9,10,11]. The symptoms of this type of psychotic disorder have been known for a long time, but it is difficult to argue that the simultaneous course of COVID-19 makes its own changes in the clinical picture of acute schizophrenia-like psychotic disorder. At the same time, most of the published works on this topic lacked detailed characteristics of the clinical picture. Mainly, they reported on the prevalence of symptoms and described the course of COVID-19 [36,44]. Few studies examined the content of psychotic experiences in psychosis, with schizophrenic symptoms occurring against the background of COVID-19 [59]. Most of the publications were represented by individual clinical cases [34,60,61]. Considering the prevalence of acute schizophrenia-like psychoses [2,3,4,5] and the high risk of developing schizophrenia [3,62], which is confirmed by the results of our study, it is important to study the features of this disorder in more detail in order to improve the care of this group of patients.

When analyzing the severity of psychopathological symptoms in acute schizophrenia-like psychotic disorder against the background of COVID-19, a high intensity of productive symptoms was revealed, represented by vivid delusional experiences with ideatory automatisms, as well as auditory hallucinations and ideas of exposure. Hallucinatory–paranoid syndrome (51%) prevailed in the structure of newly emerging acute schizophrenia-like psychotic disorder against the background of COVID-19; delusional disorder (persistent) (23%), hallucinatory (17%), and paranoid (10%) were less common. The main feature of the psychotic disorder occurring against the background of COVID-19 was the inclusion of the pandemic topic, regardless of the actual infection. Thus, in studies by other authors, the topic of the pandemic was included in the psychotic experiences of patients with a psychotic disorder in 39–58% of cases, especially in the first weeks of the spread of the virus, but afterward, there was a decrease in this indicator [63,64]. In the only review of a series of cases comparing psychoses with and without COVID-19, it was noted that the pandemic was less often included in the content of psychotic experiences in infected patients (30% vs. 78%), which differed from the results of our study [59]. The pandemic topic was most often identified (78%) in the structure of the delusional disorder (persistent) syndrome, while it played the dominant role in antagonistic delusions, delusions of grandeur, and the development of a oneiroid state (dream-like dissociative consciousness). This was followed by paranoid syndrome, in which the topic of COVID-19 was involved in the discourse of experiences in 55% of cases. Mainly, delusions of persecution with the addition of somatic delusions with abnormal tactile perceptions and visceral hallucinations were observed. In hallucinatory paranoid syndrome, the COVID-19 topic was manifested in almost a quarter of cases (21%) and was mainly characterized by delusions of exposure with the addition of sensory and motor automatisms. Only 14% of patients with a predominance of hallucinatory syndrome had the topic of COVID-19 in their psychotic experiences.

In this study, the average age of the patients diagnosed with acute schizophrenia-like psychotic disorder during the course of the pandemic was 28.5 years old (SD = 6.3). This figure differed from the results of other studies. For instance, in one of the earliest reviews of such psychoses, the average age was found to be 43.9 years old (SD = 11.8) [46]. In a separate review, it was 46.5 years old (SD = 12.1) [59], and in another review, it was 43.4 years old (the mean deviation is not specified) for men and 40.3 years for women [44]. Therefore, the average age of the test subjects in this study was considerably lower than that reported in the aforementioned reviews. However, it was similar to the average age of onset of acute psychotic disorders, which, according to the existing literature, ranged from 35.6 [65] to 37.4 years old [2]. It was hypothesized that the discrepancy in age between the current study’s group and the cited literature was explained by the inclusion criteria used in the aforementioned articles. In particular, the study groups in these articles comprised patients who had previously consulted a psychiatrist, whereas this study’s inclusion criteria excluded individuals who had previously visited a psychiatrist. Furthermore, it is important to note that, after a 36-month period, the diagnosis was changed in almost 70% of cases to schizophrenia or schizoaffective disorder. This observation allows us to make a retrospective statement that the acute schizophrenia-like psychotic disorder was a manifestation of the schizophrenic process. Hence, the average age of schizophrenia manifestation (i.e., the onset of psychotic disorders) was 27.9 years old [66], and the median age of the first psychosis episode was 28.4 years old [67].

The existing review articles on the topic of psychosis in relation to COVID-19 have mainly focused on the prevalence of psychotic symptoms. For instance, the incidence of delirium has been reported to be between 92 and 95% of cases, while disorganized behavior has been recorded in 46–48% of cases [46,59]. However, the systematic reviews included in this study encompassed cases of acute psychotic episodes in patients with a past medical history of psychoactive drug dependence or depression, which required psychiatric follow-up. In contrast, this study did not include such patients. Furthermore, this study did not consider psychosis in terms of the prevalence of symptoms in its structure, but rather in terms of the changing topic of psychotic experiences. In this study, we outlined a review study in which the authors examined the inclusion of the pandemic topic in the content of psychotic experiences [59]. This study showed that, in cases of psychosis associated with COVID-19, the pandemic topic was observed in only half the number of cases compared to cases not associated with the virus. The findings yielded somewhat contradictory data. In patients without a past medical history of severe acute respiratory syndrome (SARS), the pandemic topic was not present at all, while in cases with a past medical history of SARS, this topic was present in 55% of observations (*n* = 122). This discrepancy can be explained by the inclusion of patients with drug addiction, as well as cases of encephalitis, drug delirium, and a combination with decompensation of chronic somatic diseases in the study. Hence, an acute schizophrenia-like psychotic disorder accounted for 17% of cases. At the same time, when analyzing individual cases, there was a degree of coincidence with the results obtained. In particular, within the auditory hallucination structure, a patient perceived threats that “her mother would die from COVID-19” [13]. In another case, the disorganization of behavior that culminated in a suicide attempt was attributed to paranoid ideation, manifesting as a fear of transmitting the virus to family members [68]. In another instance, a patient’s behavior was attributed to delusional disorder (persistent), characterized by the belief that the pandemic could be prevented by saving the world [69]. And in one more case, a patient attributed the pandemic to his own actions, believing them to be the cause [42]. It should also be noted that, at the beginning of the pandemic, this topic was recorded in more than 50% of cases, though this figure subsequently decreased [63,64]. This decrease may be related to the habituation of quarantine conditions and the need to cope with everyday activities.

Psychopharmacotherapy received against the background of COVID-19 has been considered mainly from the point of view of the interaction of psychotropic and antiviral drugs. That is, in the published works, only aspects of drug interaction have been studied [28,70]. For example, a high risk of adverse events has been reported with a combination of hydroxychloroquine and haloperidol, moderate for hydroxychloroquine and risperidone or olanzapine with azithromycin, low for hydroxychloroquine and aripiprazole [28]. However, it should be noted that new data on COVID-19 therapy, which was initially recommended for administration, has been published after the pandemic [71,72]. Another area of study of drug effects in psychosis and COVID-19 is the variability of drug bioavailability. Bioavailability and distribution of drugs have been shown to be altered due to liver dysfunction [29], associated with the development of a systemic inflammatory response [73], as well as when quitting smoking [74]. Not everything was known about the ability of antiviral and psychotropic drugs to reduce or enhance the effectiveness of each other. It seemed difficult to predict their interaction with CYP3A4 inhibitors/inducers [75]. Thus, there are no generally accepted recommendations for the treatment of acute schizophrenia-like psychotic disorder occurring against the background of COVID-19. According to the data from individual clinical cases, atypical antipsychotics were mainly used [31,32,33,34,35] in minimal or average therapeutic doses [36,37,38], and in some cases, in the first days of admission, haloperidol was used in an injectable form, followed by a switch to a second-generation antipsychotic [76,77,78]. In some cases, benzodiazepines were prescribed in combination with antipsychotics [20,39] and mood stabilizing agents [40,41]; electroconvulsive therapy was used to enhance the effect of drug therapy and overcome resistance [42,43]. In some published cases, antiviral therapy was not performed at all, despite the detected infection [31,79,80]. The results of our study were consistent with the literature data on the antipsychotic drugs used. However, specific features were found in their dosage in patients with COVID-19. The dosages used were therapeutic, but they were significantly higher in patients with acute schizophrenia-like psychotic disorder and infection. We assume that this is due to the greater intensity of productive symptoms in this group of patients, which was shown by us in the first part of the study. It also cannot be excluded that an increase in the doses of antipsychotic drugs is associated with their simultaneous use with antiviral drugs, but there is no definitive evidence to confirm this. Changes in the concentration of antipsychotics when taken concomitantly with antiviral drugs (it is a reminder that in our study, umifenovir was used in 28% of cases, hydroxychloroquine in 28%, favipiravir in 21%, and imidazolyl ethanamide pentandioic acid in 18%) have not yet been thoroughly studied, and the data obtained are contradictory. For example, in a review on the interaction of these groups of drugs, it has been reported that favipiravir should be prescribed with caution only with chlorpromazine and quetiapine [81], and no changes in the concentration of antipsychotics have been reported when taking umifenovir. The interaction of hydroxychloroquine with antipsychotics has been studied more thoroughly. For example, it has been shown that hydroxychloroquine can slightly inhibit CYP2D6 metabolism (in this study, this is important when using haloperidol, risperidone, and aripiprazole), but there were great concerns about the possible prolongation of the QT interval during their interaction [82]. At the same time, the antiviral effect of antipsychotic drugs has been reported, which may also contribute to the distribution of the drug in a patient’s body [83,84]. Given the published data on the interaction of drugs with each other and the spectrum of action of antipsychotics, the interpretation of the data we have obtained is far from a final decision, since it is necessary to continue studies in a given direction.

Whilst this study focuses on the clinical presentation, treatment, and outcome of acute schizophrenia-like psychotic disorder in COVID-19, the likely triggering factors that contribute to the development of psychosis should be highlighted. It is challenging to differentiate between the direct impact of an exogenous factor (in this case, the virus itself) and the stress that was necessarily associated with the pandemic, such as strict lockdown measures, a fear of infection, a loss of stable income, the death of loved ones, and many others [85]. It has been shown that these factors could lead to an increased risk of developing anxiety, anger, and other psychological symptoms [86]. For instance, the rate of depressive disorder increased during the first year of the pandemic (682.4 [574.1–807.2] new cases per 100,000 population, representing a 27.6% [25.1–30.3] increase) [87]. However, it should be noted that, after one year, these data were considered somewhat overestimated, since most people demonstrated resilience and psychological adaptability [88], and after a certain period, the study showed that mental health levels were similar to pre-pandemic levels [89]. The existence of such contradictions gives rise to two conclusions that require further study. First, the stress factor of the pandemic is related to the duration of its impact on humanity (the longer and more habitual it is, the more people adapt). Second, given the increase in stress at the beginning of the pandemic, an increase in psychotic episodes would be expected, since it has long been shown that stress increases the risk of developing psychosis [90]. Nevertheless, the second viewpoint has not been justified, which is confirmed by longitudinal clinical follow-up. The true contribution of COVID-19 exposure to the development of psychosis remains to be definitively established [91]; it has been shown that the development of schizophrenia may be influenced by certain infections, especially those acquired during the early stages of life [92]. It is also important to note that the ability of the SARS-CoV-2 virus to disrupt the properties of the blood–brain barrier and cause psychiatric symptoms has been proven [47,93,94]. Therefore, in this study and in general studies of psychotic disorders associated with COVID-19, it is difficult to differentiate between the contribution of exogenous damage and stressors. It can be assumed that both exogenous stress and trauma contribute to the development of psychosis. However, the relative contribution of these factors remains to be determined.

Studies on clinical and follow-up analysis have shown a favorable outcome of acute schizophrenia-like psychotic disorder against the background of COVID-19. However, such studies are few and limited to several months of follow-up [36,40,44,95,96]. At the same time, if we consider an acute schizophrenia-like psychotic disorder without taking into account the effect of such an exogenous factor as infection, this diagnosis of less than a third of patients with an acute schizophrenia-like disorder (F23.2) was retained according to the results of prospective studies [4,97,98]. The results of our study confirmed these findings. Thus, regardless of the presence or absence of an acute respiratory viral infection at the onset of psychotic disorders, the outcome of the disease does not differ from the data obtained earlier in other studies. To summarize, it is legitimate to revise this type of psychosis in the new version of the ICD-11, where this disorder is transferred from the group of transient psychoses to the title “Unspecified primary psychotic disorder” in the category “Schizophrenia and other primary psychotic disorders” [8].

## 5. Limitations

This study was subject to several limitations. First, the study of changes in the clinical presentation of acute schizophrenia-like psychotic disorder did not take into account correlations with the severity of the disease, its phase of infection, and specific drugs. It is acknowledged that the study did not include patients receiving glucocorticosteroids or oxygen therapy, who might develop psychotic disorders. Furthermore, it is crucial to highlight that the impact on the clinical manifestation of psychosis was not directly dissociated from the virus, the organism, or the stressors associated with the pandemic. The findings of this study contradicted the existing literature and required further replication or refutation. The findings of this study were constrained by the limited sample size and the absence of a discernible pattern of the course of psychosis in the context of the impact of the novel virus. Another limitation of this study was the absence of analysis, a definitive understanding of the contribution of stress factors (associated with the pandemic and isolation), and the impact of the viral agent on the reported changes in clinical presentation and treatment.

## 6. Conclusions

Thus, the findings of the study allowed us to establish the instability and variability of the diagnosis of an acute schizophrenia-like psychotic disorder, irrespective of the presence of an acute respiratory infection. Over a period of three years, the diagnosis of an acute psychotic disorder similar to schizophrenia transformed into a chronic course, predominantly manifesting as schizophrenia or schizoaffective disorder. It is extremely important to continue a longitudinal clinical follow-up of acute schizophrenia-like psychoses that manifest themselves against the background of exogenous factors, such as infections, in order to improve the possibilities of differential search, personalized optimization of therapy, and identification of risk groups for an adverse outcome. Furthermore, there is a necessity for psychiatrists to collaborate with research scientists to optimize drug prescriptions. Acute schizophrenia-like psychotic disorder is a condition that deserves special attention from clinicians and researchers, as it is at this stage that patients first encounter psychiatric services. And it is here where their therapy adherence can be developed.

## Figures and Tables

**Table 1:** General characteristics of patients with acute schizophrenia-like psychotic disorder against the background of COVID-19 and without COVID-19.

General Characteristics	Group I F23.2 with COVID-19, *n* = 222	Group II F23.2 Without COVID-19, *n* = 88	*p*/χ^2^ (*p*)
Age, years ± st. dev.	28.5 ± 6.3	27.5 ± 6.3	0.022
Gender, *n* (%)			
Male	98 (44)	37 (42)	0.113 (0.737)
Female	124 (55)	51 (58)	
Education, *n* (%)			
Graduated from university	116 (52)	39 (44)	2.26 (0.521)
Dropped out of the 2–3 years of the university	34 (15)	18 (20.5)	
Graduated from college	46 (21)	18 (20.5)	
Graduated from high school	26 (12)	13 (15)	
Duration of education, years ± st. dev.	14.3 ± 2.2	13.9 ± 2.4	0.138
Employment, *n* (%)			
Employed	89 (40)	10 (11)	34.0 (<0.001)
Student	33 (15)	7 (8)	
Non-employed	99 (44.5)	71 (81)	
Retiree	1 (0.5)	0	
Marital status, *n* (%)			
Marriage	53 (24)	13 (15)	3.78 (0.286)
Single	151 (68)	66 (75)	
Divorced	17 (8)	9 (10)	
Widow/widower	1 (0.5)	0	
Scale indicators of Positive and Negative Syndrome Scale (PANSS), av. score ± st. dev.			
Summary	101 ± 19.0	104 ± 14.7	0.166
Positive subscale	32.7 ± 5.0	29.3 ± 3.9	<0.001
Negative subscale	21.7 ± 7.1	25.4 ± 3.9	<0.001
General subscale	46.9 ± 11.6	49.7 ± 11.3	0.053
P1, delusions	6.2 ± 0.7	5.6 ± 0.8	<0.001
P2, conceptual disorganization	5.9 ± 0.8	5.2 ± 0.5	<0.001
P3, hallucinations	5.5 ± 1.5	5.1 ± 0.8	<0.001
P5, grandiosity	3.6 ± 1.9	2.4 ± 1.5	<0.001

**Table 2:** Psychopharmacotherapy used to treat psychotic disorder among two groups of patients with acute schizophrenia-like psychotic disorder.

	Group I F23.2 with COVID-19, *n* = 222		Group II F23.2 Without COVID-19, *n* = 88		*p*
Antipsychotics					
Drug	*n* (%)	Mean ± SD (mg/day, CPZ eq. *)	*n* (%)	Mean ± SD (mg/day, CPZ eq. *)	
Haloperidol	104 (47)	15.2 ± 4.1 (305 ± 83)	50 (57)	10.9 ± 3.6 (218 ± 72.0)	**<0.001**
Zuclopenthixol	44 (20)	68.2 ± 24.3 (273 ± 97.3)	21 (24)	54.8 ± 15 (219 ± 60.2)	**0.025**
Risperidone	36 (16)	6.3 ± 0.8 (475 ± 56.7)	10 (11)	5.2 ± 1 (390 ± 77.5)	**<0.001**
Chlorpromazine	16 (7)	125 ± 70.1 (125 ± 70.1)	3 (3)	75 ± 0 (75 ± 0)	0.248
Olanzapine	16 (7)	20 ± 0 −600	2 (2)	10 ± 0 −300	NaN
Ziprasidone	6 (3)	63.3 ± 8.2 (158 ± 20.4)	2 (2)	60 ± 0 (150 ± 0)	0.773
Chlorpromazine equivalent of antipsychotic drugs					
Average values in the group, mg/day	330 ± 139		233 ± 92.2		**<0.001**
Anticholinergic antiparkinson agents					
Trihexyphenidyl	69 (31)	5.5 ± 0.8	36 (41)	5.3 ± 1	0.105
Biperiden	61 (28)	5.8 ± 0.7	17 (19)	5.6 ± 0.8	0.326
Benzodiazepines					
Diazepam	42 (19)	10 ± 0	56 (64)	10 ± 0	NaN
Mood stabilizer					
Valproic acid	60 (27)	1591 ± 393	27 (31)	1611 ± 376	0.831

*—Chlorpromazine equivalent. Statistically significant indicators are highlighted in bold.

**Table 3:** Change in diagnosis after 36 months of follow-up among patients with acute schizophrenia-like psychotic disorder at the onset of the disease.

Diagnosis (ICD-10)	Group I F23.2 with COVID-19, *n* = 127, *n* (%)	Group II F23.2 Without COVID-19, *n* = 76, *n* (%)	χ^2^ (*p*)
Schizophrenia (F20)	49 (39)	35 (46)	7.20 (0.066)
Schizoaffective disorder (F25)	25 (20)	21 (28)	
Bipolar affective disorder (F31)	6 (5)	-	
Lost to psychiatric follow-up	47 (37)	20 (26)	

**Table 4:** Clinical and dynamic indicators for two groups of patients after 36 months of follow-up.

Parameter	Group I F23.2 with COVID-19, *n* = 127, *n* (%)	Group II F23.2 Without COVID-19, *n* = 76, *n* (%)	*p*/χ^2^ (*p*)
Clinical and dynamic indicators			
Average number of hospital admissions in 36 months in a 24-h hospital, times	1.9 ± 1.1	1.8 ± 1.1	0.643
The average number of admissions for 36 months in a day hospital, times	1.7 ± 0.8	1.7 ± 1.0	0.666
They were sent to a day hospital, *n* (%), of them for:	71 (56)	46 (61)	22.5 (1000)
exacerbations of positive symptoms	37 (29)	21 (28)	**6.94 (0.031)**
joining depressive and anxiety symptoms	11 (9)	5 (7)	
increases in negative symptoms	23 (18)	2 (26)	
Occupational disability, *n* (%)	34 (27)	22 (29)	0.113 (0.737)
Maintained employment without role demotion, *n* (%)	21 (17)	1 (1)	4.46 (0.108)
Reduced qualifications, *n* (%)	24 (19)	1 (1)	
Stopped working, *n* (%)	31 (24)	7 (9)	
Continued their studies, *n* (%)	12 (9)	4 (5)	0.183 (0.669)
Stopped studying, *n* (%)	13 (10)	3 (4)	
Scale, mean ± SD			
Positive and Negative Syndrome Scale (PANSS)	58.6 ± 21.9	60.5 ± 16.1	0.1
PANSS P	10.2 ± 7.3	11.3 ± 9.2	0.242
PANSS *n*	29.0 ± 7.1	31.5 ± 7.2	**0.003**
PANSS G	19.6 ± 9.5	17.8 ± 7	**0.005**

Statistically significant indicators are highlighted in bold.

## Data Availability

The data presented in this study are available on request from the corresponding author due to privacy and legal reasons.

## Abbreviations

The following abbreviations are used in this manuscript:

ICD-10	International Classification of Diseases 10th Revision
ICD-11	International Classification of Diseases 11th Revision
CPZ eq.	Chlorpromazine equivalent
COVID-19	Coronavirus disease 2019
